# Deep Ensemble Fake News Detection Model Using Sequential Deep Learning Technique

**DOI:** 10.3390/s22186970

**Published:** 2022-09-15

**Authors:** Abdullah Marish Ali, Fuad A. Ghaleb, Bander Ali Saleh Al-Rimy, Fawaz Jaber Alsolami, Asif Irshad Khan

**Affiliations:** 1 Department of Computer Science, Faculty of Computing and Information Technology, King Abdulaziz University, Jeddah 21589, Saudi Arabia; 2 Faculty of Engineering, School of Computing, Universiti Teknologi Malaysia, Johor Bahru 81310, Malaysia; 3 Department of Computer Engineering and Electronics, Sanaá Community College, Sanaá 5695, Yemen

**Keywords:** fake news detection, misinformation, two-stage classification, deep learning, ensemble model

## Abstract

Recently, fake news has been widely spread through the Internet due to the increased use of social media for communication. Fake news has become a significant concern due to its harmful impact on individual attitudes and the community’s behavior. Researchers and social media service providers have commonly utilized artificial intelligence techniques in the recent few years to rein in fake news propagation. However, fake news detection is challenging due to the use of political language and the high linguistic similarities between real and fake news. In addition, most news sentences are short, therefore finding valuable representative features that machine learning classifiers can use to distinguish between fake and authentic news is difficult because both false and legitimate news have comparable language traits. Existing fake news solutions suffer from low detection performance due to improper representation and model design. This study aims at improving the detection accuracy by proposing a deep ensemble fake news detection model using the sequential deep learning technique. The proposed model was constructed in three phases. In the first phase, features were extracted from news contents, preprocessed using natural language processing techniques, enriched using n-gram, and represented using the term frequency–inverse term frequency technique. In the second phase, an ensemble model based on deep learning was constructed as follows. Multiple binary classifiers were trained using sequential deep learning networks to extract the representative hidden features that could accurately classify news types. In the third phase, a multi-class classifier was constructed based on multilayer perceptron (MLP) and trained using the features extracted from the aggregated outputs of the deep learning-based binary classifiers for final classification. The two popular and well-known datasets (LIAR and ISOT) were used with different classifiers to benchmark the proposed model. Compared with the state-of-the-art models, which use deep contextualized representation with convolutional neural network (CNN), the proposed model shows significant improvements (2.41%) in the overall performance in terms of the F1score for the LIAR dataset, which is more challenging than other datasets. Meanwhile, the proposed model achieves 100% accuracy with ISOT. The study demonstrates that traditional features extracted from news content with proper model design outperform the existing models that were constructed based on text embedding techniques.

## 1. Introduction

Fake news, also called misinformation, is generated by many actors, including organizations and individuals. It is created to: drive sales by glorifying specific products and disseminating negative views of competitors’ products, gain political benefits such as directing elections, make financial gains, maintain life quality, and so on [1,2]. Recently, fake news has been propagated in huge figures due to the use of social media and the imposed lockdown caused by COVID-19 [3]. The Internet has become the primary medium for communication, business activities, and services. Fake news causes harmful impacts on society, stability, or on targeted individuals [4]. According to Ansar and Goswami [2], social media have become the main source of news for many individuals, and it is the most preferred medium for sharing fake news among individuals [5,6,7,8]. Once false news or misinformation is shared on social media platforms, it propagates virally faster than true news due to the attraction of the novelty of false news [9]. For example, in 2016, false news related to the U.S. president was shared 30 million times in Trump’s favor compared to 7.6 million times that fake news was shared favoring Hillary Clinton [2,10]. Moreover, recently, false information related to COVID-19 has seriously confused many people around the world regarding the reality of the pandemic as well as the effectiveness of the vaccine [11]. According to Zaryan [12], fake news attracts readers more readily than genuine news. Accordingly, fake news spread very quickly, became more popular, and had a deeper impact [10,13]. Because social networks are the major source of fake news and the platforms where fake news is proliferated, many service providers such as Google, Twitter, and Facebook are alerted to controlling the spread of fake news by finding accurate solutions to automatically detect fake news before it can spread.

Moreover, a few websites try to check news authenticity, such as FactCheck, the Washington Post Fact Checker, and PolitiFact, to mention some [14]. However, fake news detection is not a trivial task that the industrial sector can make. Fake news detection needs concerted efforts between research and service providers for subtlety and quick detection.

Accurate detection of fake news is challenging due to the following reasons. Fake news shares similar linguistic features with real news [1]. Most of the fake news is a fabrication of real news; thus, it is difficult to identify correct news content. Fake news is domain-dependent which needs domain experts to annotate a large amount of data for supervised learning [2,15]. Detecting fake news is a complex task due to the need for multiple drip lines, including machine and deep learning, data science, features engineering, statistics, journalism, psychology, and social science. According to Long [15], a model train for detecting fake political news is ineffective for fake health care news. Accordingly, deep learning models trained on large datasets collected from multiple domains are required. However, many remaining issues need to be addressed to improve detection performance.

The incentive for our research is to propose accurate detection models to detect false news to curb the dissemination of misinformation. Many issues have been investigated, such as which linguistic features are more effective, feature representation, classification method, and model design. Different types of features related to the content [14,16], source [17], social response [18], and news context [19] were extracted and investigated. The content-based features were the most studied and used [20]. Many text representations were proposed, including the bag-of-words (BoW) model such as term frequency–inverse document frequency (TF–IDF) [21], the Bidirectional Encoder Representations from Transformers (BERT) [22], word embedding techniques such as word2vec [23], GloVe [24] and Funnel [22]. The pre-trained language models such as GloVe, BERT, and Funnel were widely used for text representation due to the inclusion of word context and semantics into the representation. Various machine and deep learning techniques were also investigated [8,16,25,26,27,28,29], such as linear super vector machine (LSVM) [15], Random Forest (RF) and decision tree [30], capsule neural networks [23], and convolutional neural network [31,32]. CNN-based classifiers were frequently used as it proves their effectiveness for text classification in different domains. Hybrid [33,34] and ensemble learning [35,36,37] have also been investigated. Many datasets were investigated, such as LIAR [31], ISOT [24], and COVID-19 [38]. Finding distinguishing patterns in some datasets is easy such as ISOT [24] and COVID-19 [38], while others, such as the LIAR dataset, are challenging for classification due to the contents of short news sentences. 

The content-based features were the most studied and used [5,6,20,39]. However, the effectiveness of such features can be reduced by fake news authors due to the ability to create fake news with high similarity to real news. In such cases, the language models widely utilized by many research studies for text understanding are unsuccessful for fake news detection, especially in the early stage of news dissemination or in the case of short news sentences. Due to the high similarity between fake and real news, especially when news is delivered in a short sentence, such as in social media posts, word embedding techniques usually either use a sparse tensor or similar genuine news patterns. Some fake news contains realistic facts in its content to make the illusion more effective [22]. Accordingly, despite being the best fit for language model representation, CNN-based models, may not have the best performance for short news sentences in which insufficient features are introduced to the model due to the sparsity problem of the features’ tensors created by the embedding technique. This is clear from the performance of the existing solutions on the LIAR datasets, which is lower than 49% detection accuracy. Therefore, there is a need to investigate a new fake news detection model to improve detection accuracy.

This study aims to design and develop a deep ensemble learning-based fake news detection model to improve the detection performance of fake news detection. The model consists of three phases. In the first phase, the features are represented using the TF-IDF technique. The semantic and word context representations were removed from the feature sets to reduce the similarity between fake and genuine news. New representative features are derived using the n-gram model. In the second phase, an ensemble of sequential and dense deep learning prediction models was designed and developed to extract the hidden and more representative features. The ensemble consists of multiple binary classifiers, each of which predicts the degree of news correctness. That is, the features that represent the news class were learned using deep learners. Such features are extracted from the last layer of the deep ensemble model. In the third phase, the score outputs of the deep learning predictors are used to train a multilayer perceptron (MLP) for the final decision. Results show that the proposed model in this study outperforms the state-of-the-art models. This study makes the following contributions.

1. Deep ensemble fake news detection model using deep learning and multilayer perceptron constructed in two learning stages. The first stage is used to extract the hidden features based on the level of the correctness of the news. The second learning stage is to learn the relationships between aggregated outputs of the ensemble deep classifiers and the target class, utilizing the hidden features extracted from the previous stage for the final decision on news type.
2. Hidden representative features were extracted by developing multiple binary classifiers based on news correctness levels, such as false, half-true, and true news. In doing so, gradual yet abstract features can be created that distinguish the representative patterns well. These features were used to train more effective classifiers. We hypothesize that the intermediate features contain hidden fake news patterns.
3. Intensive experiments were conducted to validate and evaluate the proposed model. The most common datasets that the state-of-the-art models use were utilized for the evaluation in this study.

The remainder of this article is arranged as follows. The related work is discussed in Section 2. Section 3 describes the suggested model, while Section 4 details the experimental methods. Section 5 contains the results and comments, and Section 6 draws the conclusion of this study.

## 2. Related Work

Many solutions have been proposed for accurate detection of fake news. Several different approaches were researched, including feature extraction, representation, classification, and model design to improve the detection performance. However, the detection of fake news detection is complex. Many issues are still open for researchers, such as improving detection accuracy, early detection of fake news in social media before it spreads, and the way by which fake news spreads. Huang and Chen [1] proposed a fake news detection model using ensemble learning. The ensemble learning consists of four classifiers, namely, embedding LSTM, depth LSTM, LIWC CNN, and n-gram CNN. These classifiers were trained based on representative features using the Word2vec embedding technique. The Self-Adaptive Harmony Search (SAHS) was used to optimize the weights of the ensemble classifiers. However, the main limitation of this model is that it was not designed for early detection or for short news statements such as used in social media. Wang [31] proposed a dataset called LIAR that contains 12.8K manually labeled short sentences. The dataset was collected from POLITIFACT.COM, accessed on 5 January 2022. Many classifiers were investigated for automatic news detection, including LR, SVM, Bi-LSTM, and CNN. The CNN model achieved classification accuracy of 27%, outperforming other tested classifiers.

Samadi, Mousavian [22] devised a model for detecting fake news using contextualized embedding and deep learning. Three classifiers were trained, namely convolutional neural network (CNN), multilayer perceptron (MLP), and single -Llayer Perceptron (SLP). Four pre-trained models, namely BERT, RoBERTa, GPT2, and Funnel, were used for training the feature representation and used as input for training the classifiers. Funnel-CNN was reported to have the highest accuracy compared to the other studies’ models. Three datasets were used for evaluating the proposed models, namely, LIAR [31], ISOT [24], and COVID-19 [38].

Shim, Lee [11] devised a fake news detection model based on URL based embedding technique. The web links that contain the news were researched, and the related features were embedded using an embedded technique derived from word2vec and called link2vec to improve the classification accuracy. Three classifiers were trained LOGIT, SVM, and ANN classifiers. Results showed that the SVM classifier outperformed the others (93.1% classification accuracy concerning the used dataset). However, deep learning classifiers were not investigated to evaluate the effectiveness of the proposed web-based embedding technique (link2vec). Moreover, this model is based on the URL where the news content is available. Thus, such a model is not suitable for detecting fake news in social media where there are no associated URLs for the news.

Nasir, Khan [34] proposed a hybrid CNN-RNN deep learning model by cascading CNN and RNN models. The proposed classifier was trained using ISOT [24] and FA-KES [40]. The news features were extracted from the datasets and embedded using the GloVe pre-trained word embedding technique. Hakak, Alazab [30] proposed an ensemble-based fake news detection model. Twenty-six features were extracted from news content. Such features include statistics about the number and average length of words, characters, and sentences. A named entity recognition algorithm was also untied to extract more statistical features related to the person, organisation, date, time, etc. Results show improvement of prediction accuracy related to state-of-the-art. However, the extracted features lead to an overfitting problem and cannot be generalized. This is clear from the gap between training accuracy (99%) and testing accuracy (44%) for short news sentences for the LIAR dataset.

Samadi, Mousavian [22] investigated different deep contextualized text representation models and proposed different deep learning classifiers. Many pre-trained models were investigated such as Funnel, GPT2, BERT, and RoBERTa. The embedding layer was connected to CNN, SLP, and MLP for classification. Results show that Funnel with CNN outperforms the state-of-the-art models. However, poor prediction accuracy was achieved in the LIAR dataset (48%).

To sum up, many techniques were explored to boost the prediction efficacy of the fake news detection model. However, detecting fake news is a complex task. Existing state-of-the-art models suffer poor detection accuracy for short news sentences. This is because embedding techniques end up with sparse feature tensors, leading to the wrong classification for novel samples. In this study, features extracted from short news were augmented with feature sequences constructed using n-gram. These features are represented using the TF–IDF technique, which excludes the semantic features, and thus reduces the number of representative features. Feature selection using information gain is used to exclude the noise and unimportant features and also to further reduce the features. Two-stage classifications are carried out based on the extracted features. The first stage consists of an ensemble of deep and dense binary classifiers, while the second stage includes a multilayer perceptron for final classification. The proposed model is further detailed in the following section.

## 3. The Proposed Fake News Detection Model

**Figure 1:**
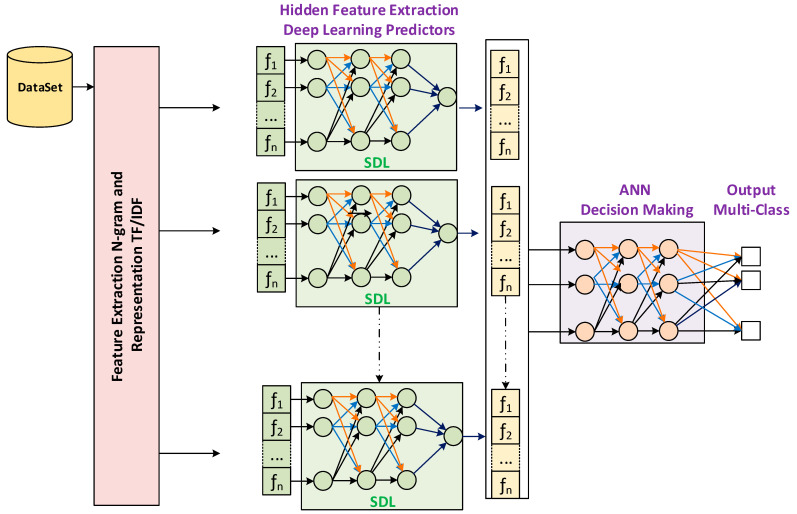
The proposed deep ensemble model.

Figure 1 shows the architecture of the proposed fake news detection model. The proposed model consists of three phases, namely, feature extraction and representation phase, ensemble deep learning classifier construction, and final multilevel perceptron classifier construction. The following subsections provide a thorough explanation of each phase.

### 3.1. Phase 1: Feature Extraction and Representation

In this phase, the features that were used to construct the proposed model were mined from different sources, including social media posts or news websites. Because news content is written in natural human language, such text usually contains abbreviations as complete words, different forms of the same verbs and nouns, and unnecessary content. Such news features increase the randomness and degrade the performance of machine learning algorithms. The removal of the undesirable features is necessary, such as the punctuations and irrelevant characters, converting the words to lower case, and normalization. The text preprocessing techniques in the natural language processing (NLP) library was utilized to preprocess the news content. Such features can impede training a precise classifier. The normalization process has two objectives. The first step is to lessen the sparsity of the feature vectors by eliminating words that aren’t essential and cutting down on the total amount of words by returning words to their original forms. The second is converting the news document or sample from unstructured form to a structured list of the unique terms in the document. The normalization process includes tokenization, removing the stop words, lemmatization, and stemming. Tokenization involves representing the news sample by a list of terms that make up the news sample. Stemming is converting the words by their roots, e.g., removing “s” from the plural nouns and removing “ing” from the verbs. In the lemmatization process, the verbs are rooted in their base form using the lexical knowledge base. For example, the verbs ‘drank’ and ‘drunk’ are converted to ‘drink’.

The n-gram technique [41] was used to enhance the set of representative features extracted from the preprocessed news text. The n-gram model, namely the bi-gram, was used in this study to reduce feature complexity. That is, each subsequent term is considered one additional feature. N-gram was widely applied for improving false news detection due to its efficacy in enhancing classification accuracy [1,23,24,26,42]. In this study, the bi-gram model was used because there was not much improvement in terms of detection performance during the experiments as compared to the tri-gram model. Accordingly, bi-gram was used for efficiency to reduce feature complexity and training time. 

A corpus containing the preprocessed terms along with their frequency of occurrence in each class was generated. Then, the words were converted to their corresponding numerical values using the statistical-based text representation technique, namely the TF–IDF. Thus, the feature vectors that represent the news samples were converted to numerical weights for deep learning learners. The TF–IDF is calculated using the following formula:

(1) $$tf-idf=tf\cdot log\frac{N}{df}\;$$

where tf denotes the term frequency, df denotes the document frequency, and N is the number of samples in the dataset. The term frequency $tf_{w}$ of a word i is the number of times a term (word) appears in the sample j divided by the number of words in the sample $d_{j}$. It can be calculated as follows.

(2) $$tf_{w}=\frac{tf_{ij}}{d_{j}}$$

Meanwhile, the inverse document frequency $\left(idf_{w}\right)$ is the logarithm of the total number of documents (samples) in the dataset divided by the number of documents that the term has occurred in:

(3) $$idf_{w}=\frac{totalnumberofsamplesinthedataset}{numberofdocumentsthat\;havethetermw}$$

The inverse document frequency is used to penalize the weights of the general terms that appear in many documents as they are less significant for the classification [43]. For example, if the word is repeated in all instances, its weight should be reduced as it is not important for the classification.

### 3.2. Phase 2: Deep Ensemble Learning

In this phase, the class label of the news datasets determines the number of classes. Because fake news may not contain pure false information, the news samples can be classified into a number of classes. Fake news usually contains true information mixed with false information. Thus, it is not easy to differentiate between true and false. Accordingly, the correctness of the information in the news can be a gradient based on how much true information is in the fake news samples, such as in the case of the LIAR dataset [31]. Therefore, multiple predictors trained based on different levels of fake information are an important step for pre-classification. These predictive learners provide the following two advantages. The first advantage is predicting the gradient of accurate information in the news sample, while the second advantage is extracting the hidden patterns representing the news label during the training. However, in the case of binary classes such as in the ISOT dataset [24], one predictive learner is constructed.

**Figure 2:**
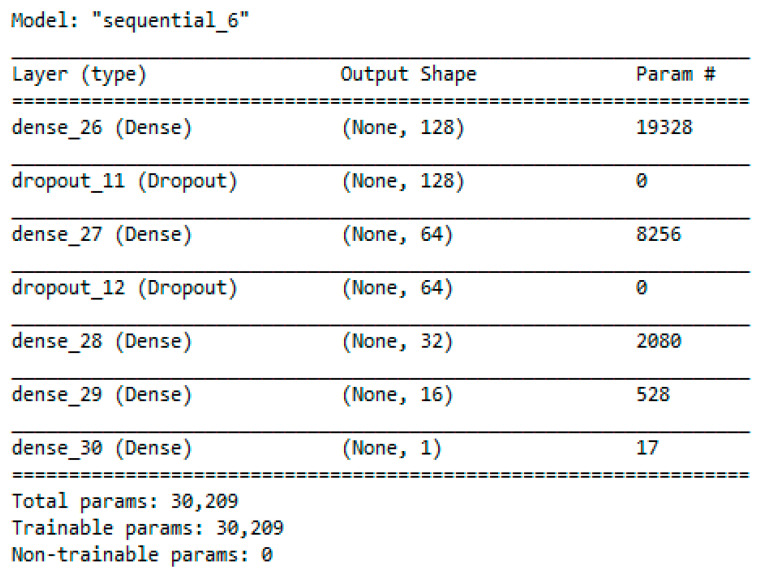
Summary of a single designed deep learner.

**Figure 3:**
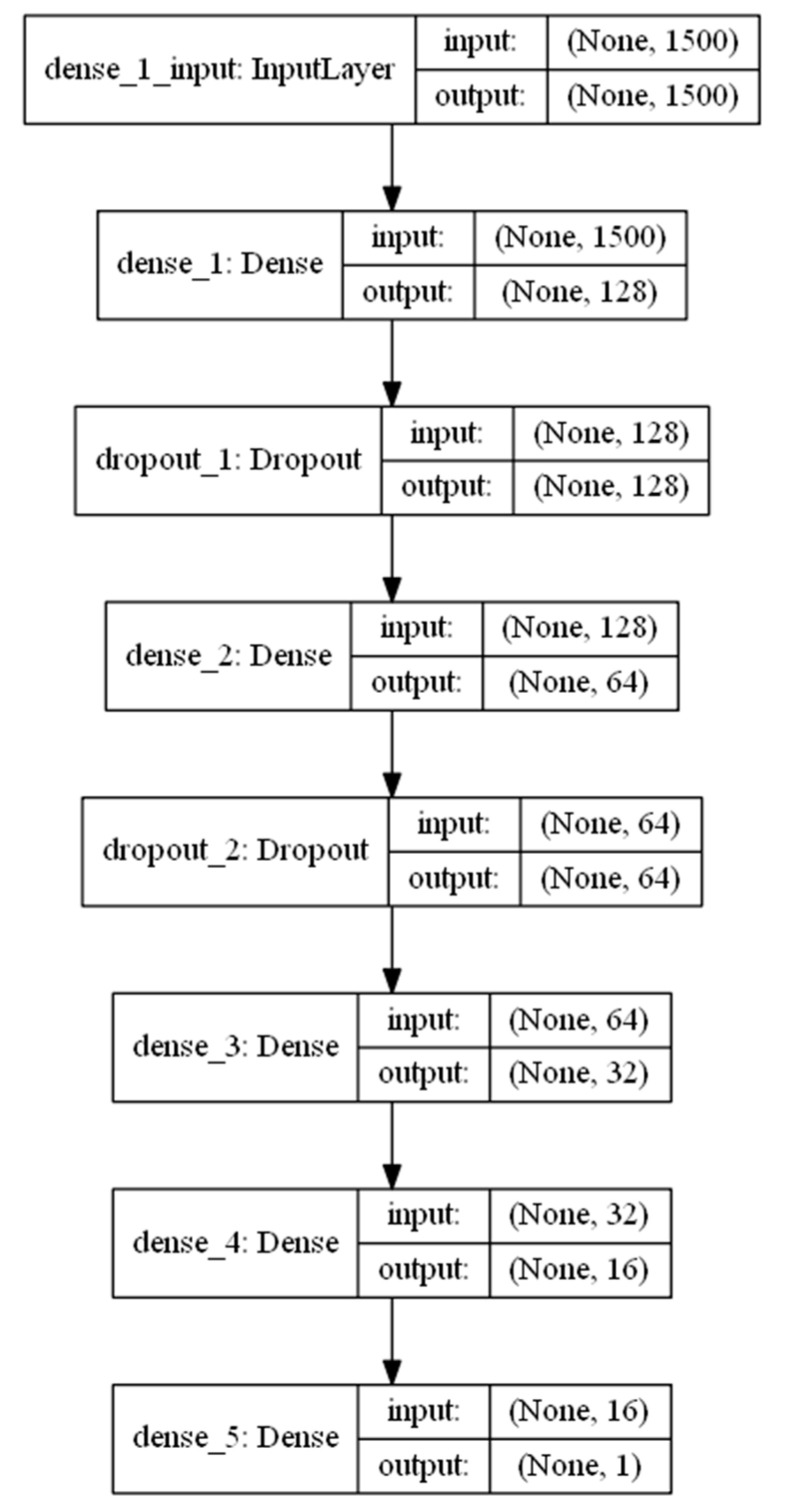
Architecture of a single designed deep learner.

Accordingly, in this phase, six deep learning models were constructed using dense sequential networks. Sequential deep learning is used to effectively capture different patterns related to different news classes, such as half-true, barely-true, or totally fake news. The hidden features of each class can be recognized and extracted [44]. Each network consists of seven layers, as presented in Figure 2 and Figure 3. Layers 1, 3, 5, 6, and 7 are dense layers consisting of 128, 64, 32, 16, and 1 neurons for each layer, respectively. Layers 2 and 4 are dropout layers for regularization and to avoid overfitting for generalization. Selection of the number of neurons in each layer is a challenging problem. However, a commonly accepted method is to select empirically. In this study, the number of neurons in the first layer was selected heuristically. In contrast, the size of the other hidden layers was obtained by dividing the number of preceding features by two, so that the abstracted hidden features were obtained gradually to increase the abstraction with high variance.

In each dense layer, the ReLu function is utilized as an activation function, and the sigmoid function is employed as a decision-making function at the output layer. The stochastic gradient descent algorithm’s modification, known as the Adam optimizer, was used to update the weights and decrease the learning error. This is the type of adaptive gradient which uses a dynamic learning rate estimated with the adaptive moment estimation technique. Such an algorithm improves the training performance of problems with sparse gradients, such as the case of short fake news sentences. After the training, the weights of the neurons of the output layer were used as new hidden features to detect the hidden fake news patterns. These weights were fed to the ultilayer perceptron for final classification.

The purpose of the ensemble set of deep learning predictors is to train multiple predictors using the deep network, as in Figure 2 and Figure 3. The aim is to extract new hidden features. These features are represented by the weights of the neurons in the deep learning network. The best parameters will give the best prediction of the hidden patterns. With $x_{i}$ as the input TF/IDF feature and $w_{ij}$ as the weight of the neuron connected to the input features in level j, the following steps were followed to train each predictor in the deep ensemble.

1. A class in the dataset represents the level of news correctness. For each class in the dataset, the class samples were set as a positive class, while other samples belonging to the other classes were set to a negative class. The aim is to extract the distinctive features that represent that class well.
2. For each new dataset created from the first step, the dataset is split into three sets: training, validation, and testing. The training set is used to learn what is the best set of weights that reduces the distance between prediction and actual instance. The validation set is used for turning the parameters. Meanwhile, the testing sets are used to evaluate the performance of the predictor.
3. The dataset samples are preprocessed using the preprocessing steps as described in the first phase and used as input to the developed deep learning model (as presented in Figure 2 and Figure 3).
4. The model parameters are initialized randomly using Xavier–Glorot initialization as follows.
  (4) $$N\left(0,\;\frac{2}{n_{i}}\right)$$
  The initialization maintains a smooth distribution of the weights by making the variance of the activations the same across the layers.
5. For N={1,2,…n} where n is the number of epochs:
  - ∀i ∈N, use the neurons to predict $x_{i}$ to produce the prediction ${\hat{y}}_{i}^{\theta }$.
  - Minimize the loss function  J(θ) to evaluate the distance between the actual values $y_{i}^{\theta }$ and predicted values ${\hat{y}}_{i}^{\theta }$ using the weights vector θ where *m* is the number of samples in the training set, ℒ is the cost function, and δ denotes the dropout rate, as follows.
    (5) $$J\left(\theta \right)=\{\begin{array}{c} \frac{1}{m}\overset{m}{\underset{i=1}{\sum }}ℒ\left({\hat{y}}_{i}^{\theta },\;y_{i}^{\theta },\delta \right)\;if\;dropout\;layer\\ \frac{1}{m}\overset{m}{\underset{i=1}{\sum }}ℒ\left({\hat{y}}_{i}^{\theta },\;y_{i}^{\theta }\right)\;without\;dropout\;layer \end{array}\;$$
  - Use the gradient descent algorithm to update the weights θ as follows.
    (6) θ=:G(θ)

After convergence, the parameters in vector θ (which contains the aggregated weights of the neuron in the last hidden layer) are used as new hidden and representative features to train the multilayer perceptron prediction model for final classification.

### 3.3. Phase 3: Multilayer Perceptron (MLP) Classification

In this stage, the MLP is constructed, being the most commonly used by researchers for classification and regression tasks. The features that were extracted from the output of the six deep predictors θ were used as input for the MLP. The MLP consists of five layers, the input layer, two hidden layers, and the output layer consisting of twelve, thirty-two, sixteen, and six neurons for multi-class classification tasks with ReLu activation functions and SoftMax functions. The number of neurons and hidden layers were determined by trial and error, and the best numbers were selected. The input features of the MLP classifier are extracted from the trained deep learning model in the previous phase as follows. Let p(c) denote the probability of predicting a specific class (e.g., fake news), then:

(7) $$p\left(c\right)=\overset{n}{\underset{i=1}{\sum }}w_{i}*x_{i}+\theta \;$$

where $w_{i},\;x_{i},\;\mathrm{and}\;\theta$ denote the weight of the neuron, the corresponding output of the previous layer, and the weights of the deep learners as trained in the previous phase, respectively. The weights $w_{i}$ are learned based on the output of the deep learning classifiers using the MLP. Each classifier contributes to computing the weights and derives the p(c). The final classification score S(c) of the news class is calculated using the sigmoid function as follows.

(8) $$S\left(c\right)=\frac{1}{a+e^{-p\left(c\right)}}$$

## 4. Performance Evaluation

In this section, the data sets are described by explaining the performance evaluation.

### 4.1. Datasets

Several datasets are commonly used by researchers to benchmark the fake news detection models. Social media such as Facebook and Twitter were the source of both fake and true news. Having been widely used by other researchers, the two common datasets, LIAR [31] and ISOT [24], were used in this study.

#### 4.1.1. LIAR Dataset

The LIAR dataset was generated by Wang [31]. It is commonly used by researchers such as [22,23,30,31,32,45] to validate their proposed fake news detection models. The LIAR dataset is publicly available (on https://www.cs.ucsb.edu/~william/data/liar_dataset.zip, accessed on 20 January 2022). It comprises 12,836 short statements that were gathered from Politifact.com (https://www.politifact.com/, accessed on 5 January 2022). The news statements that are in Politifact.com were originally collected from different sources such as TV advertisements, Facebook posts, Twitter, interviews, political debates, etc. The statements in the dataset are categorized by Politifact.com into six classes which are false, pants-fire false, true, half-true, mostly-true, and barely-true. In addition, the LIAR dataset encompasses details on the subject(s), including the author, the title of the author’s job, the place of residency, their political affiliation, the total number of credit record counts of each class by the author, and the context in which the statements were written.

The following is an example of news extracted from the LIAR dataset:

**“Statement:** Health care reform legislation is likely to mandate free sex-change surgeries. **Class:** false, **Subject**: Health-care, **Speaker:** blog-posting, **speaker’s job title:** NaN, **state info**: nan, **party affiliation**: nan, **context:** a news release”

**Figure 4:**
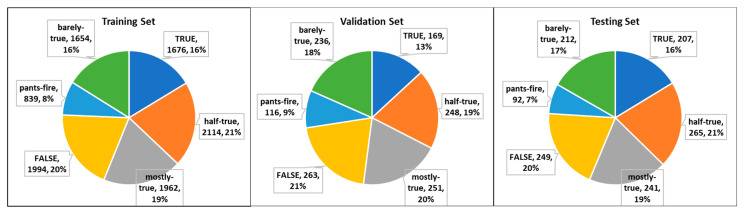
Samples distribution of the LIAR datasets.

The dataset includes the history containing several past news statements by the news writer, curated as half-true, pants-fire false, barely-true, mostly-true, false and true. The LIAR dataset consists of three groups: training, validation, and testing. Figure 4 and Table 1 contain the number of instances in each set.

#### 4.1.2. ISOT Dataset

**Figure 5:**
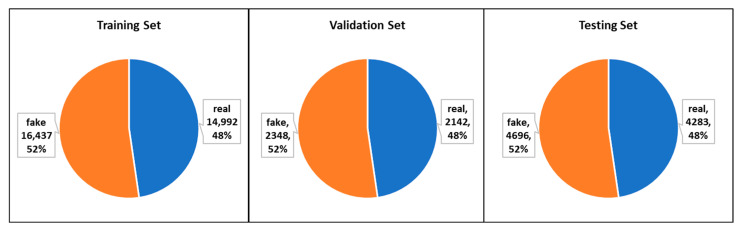
Samples distribution of the ISOT datasets.

ISOT datasets were collected by Ahmed, Traore [24], from genuine news articles. The real news was gathered from Reuters.com, accessed on 5 April 2022, while false information was gathered from unreliable sources. The dataset includes 44,898 news statements in total, 21,417 of which are genuine and 23,481 are false (see Figure 5 and Table 2). The ISOT Dataset was used by many researchers [22,23,30,32], notably for classifying long-length news articles.

### 4.2. Performance Evaluation

In this study, accuracy, precision, recall, and F1-Measure were used to validate the proposed model. These measures are commonly used in related work [22,30] and can be calculated as follows.

(9) $$\mathrm{Accuracy}=\frac{Totalnumberofsampleswhicharecorrectlyclassified}{Totalnumberofsamples}$$

(10) $$\mathrm{Precision}\;=\frac{Totalnumberofpositivesampleswhicharecorrectlyclassified}{Totalnumberofpositiveclassifiedsamples}$$

(11) $$\mathrm{Recall}=\frac{Totalnumberofpositivesampleswhicharecorrectlyclassified}{Totalnumberofpositivesamples}$$

(12) $$F1-\mathrm{Score}=\frac{2\times \mathrm{Precision}\;\times \mathrm{Recall}}{\mathrm{Precession}+\mathrm{Recall}}$$

The proposed model was evaluated by comparison with the results obtained by the following state-of-the-art models [15,22,23,24,30,31,32]. Some of these models used both LIAR and ISOT datasets such as [15,22,23,24,30,31,32], while others either used LIAR [15,22,23,30,31,32] or ISOT such as [22,23,24,30,32]. Different machine learning and deep learning techniques were used for the classification such as RF [30], LSVM [24], CNN [22,30,32,34,46], LSTM [15,47,48], and Capsule Neural Network [23].

## 5. Results and Discussion

**Figure 6:**
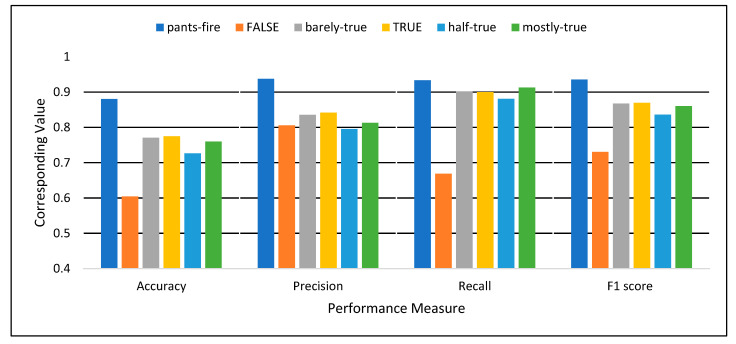
The Performance of the Multiple Binary Classification (1st Stage).

**Figure 7:**
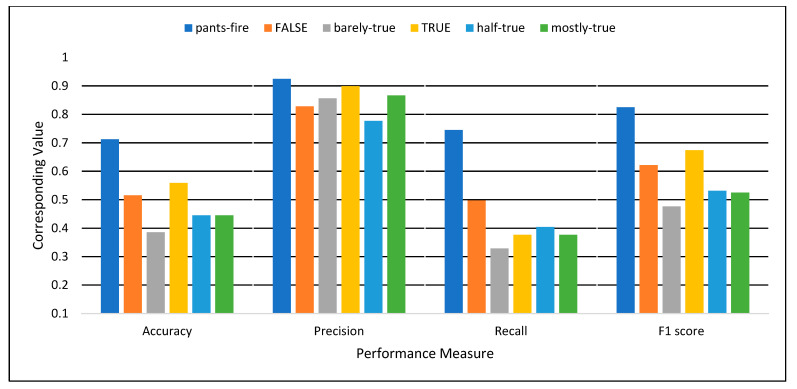
The Performance of the Multi-class Classification (2nd stage).

The experiments in this study were conducted on a computer with 4-CPUs, i7@2.5 GHz, and 8 GB RAM. The programming language Python 3.7 was used to implement the proposed model. Table 3 and Figure 6 and Figure 7 show the classification performance achieved by the proposed model. Table 3 presents the performance in terms of Accuracy, Precision, Recall, and F1 score for both first and second stage classification. Figure 6 illustrates the performance of the first stage binary classification, while Figure 7 shows the performance of the second stage, multi-class classification for decision making.

As shown in Table 3 and Figure 6, in the first stage, which consists of multiple binary classifications, each deep learning classifier can individually recognize the true class of the news with 85% overall detection performance in terms of F-score. In the case of binary classifications, the classifiers have been trained based on a single class label against the other class labels as the second label. These results indicate the effectiveness of the proposed deep learning model in classifying one type of news against the other types. However, there will be confusion when multiple classifiers output the same results for a single sample. This is why the second stage is necessary to solve the contradictions between the multiple classifiers.

It can be observed from Figure 7 and Table 3 that the second stage multi-class classifier achieved 51%, 86%, 45%, and 61% for overall accuracy, precision, recall, and F1, respectively. The highest accuracy was 71% for the pants-fire class, while the lowest achieved accuracy, 39%, was for the barely-true class. For multiple binary classifiers, the average accuracy is 75%, with an 85% F1 score (see Table 3 and Figure 4). Similarly, the pants-fire classifier achieved the best accuracy of 88%, while the half-true classifier achieved the worst accuracy. Table 3 shows that in the first stage, the binary classifiers work better than the second stage classifier. However, the final decision on binary classifiers is challenging because multiple classifiers can have the same results. It is difficult to determine which output is correct. This is why the second classifier is important for decision-making. Thus, the final classification results were obtained when the 1st stage classifiers were used to extract the hidden features and train the 2nd stage classifier. The results of the proposed model were compared to the related work, which uses the LIAR dataset to construct their models.

**Figure 8:**
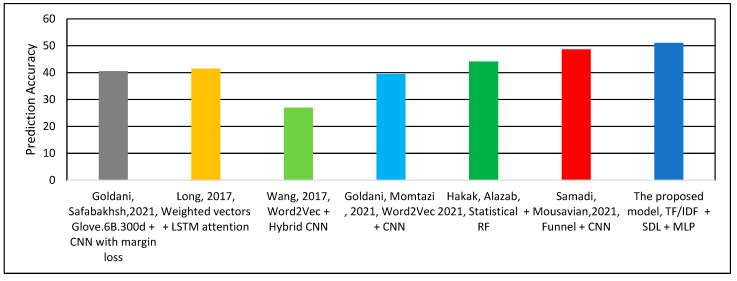
Classification Accuracy of the Models using LIAR Dataset.

Table 4 and Figure 8 show the accuracy performance of the compared models. As can be observed, the proposed model outperforms the related works. It achieves a 2.41% improvement compared to the best accuracy achieved in the related work by Samadi, Mousavian [22]. It can be observed from Table 4 that the TF–IDF and n-gram are more effective than the alternative techniques [15,23,31,32] for fake news detection. 

Table 5 shows the classification performance when applied to the ISOT dataset. As shown in Table 5, 100% was attained in the second stage for all performance measures. Meanwhile, in the 1st stage, the proposed model achieves 99.94% with respect to all performance measures. As mentioned earlier, the ISOT dataset is not challenging because it contains long text news with consistent classes. Compared with the LIAR dataset, which is more challenging, most of the machine learning techniques, both deep and conventional classification techniques, achieve an accuracy of higher than 90%. 

**Figure 9:**
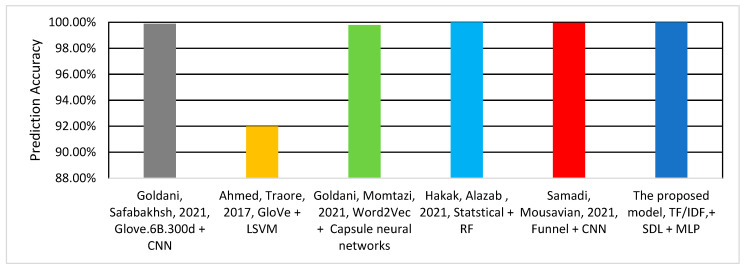
Classification Accuracy of the Models using ISOT Dataset.

Table 6 and Figure 9 show the performance comparison between the proposed model and the related work using the ISOT dataset, which has been included in this study to evaluate the performance of the proposed model on a different dataset that contains longer sentences compared to the LIAR dataset. In addition, ISOT dataset is commonly used by the related work to benchmark the fake news detection solutions {Goldani, 2021 #40; Samadi, 2021 #25; Goldani, 2021 #48; Goldani, 2021 #62; Hakak, 2021 #59}. As can be observed, the proposed model achieved 100% prediction accuracy. Similarly, the model proposed by Hakak, Alazab [30] achieves 100% prediction accuracy using the derived feature related to words and NER statistics with an RF classifier. However, it performs worse in the LIAR dataset, with only 44.15% accuracy. Although both ensemble models—the proposed model and Hakak, Alazab [30]—achieve 100% detection accuracy, the proposed model outperforms all other related models in terms of detection efficiency. That is, the proposed model is less memory intensive compared to the others model. This is because the size of the features vector extracted by the proposed model is smaller than that in the related works, which yields to faster processing and detection.

In general, the models trained using the ISOT dataset detect fake news remarkably well compared to those trained based on the LIAR dataset for two main reasons. The first is that the initial classification task was binary where two pure news types are presented in the datasets as either pure true or pure false. It is well known that binary classification is easier than the multi-class classification task [22]. The second reason is that the ISOT dataset contains long sentences which implies more distinguished features presented in the dataset. 

In summary, when we rely solely on the textual features extracted from the news, fake news detection is challenging due to the high similarity with real information and insufficient features. Moreover, extracting news features is expensive, and it is not a trivial task as it is subject to noise and fabrication for many reasons—political, racist, and financial, among many others. Even a trusted entity can become suddenly distrusted on some occasions, intentionally or unintentionally. Many researchers have previously focused on improving text classification performance by enhancing the type of features extracted from the syntax, and semantics extracted from the content or/and the context. However, the classifiers and model design types have not been deeply investigated. This study shows that the features extracted solely from the news content with proper representation and proper model design outperform the existing text embedding techniques with other classification techniques in the fake news detection domain. This is because fake news shares linguistic features with real news [1]. Accordingly, feature embedding techniques may not be the best for fake news detection as it does not necessarily contain semantic or sentiment patterns that are distinguishable from real news. Even humans tend to believe or not dispute the correctness of fake news due to its complex phenomena and involvement of many factors such as emotions, political directions, etcetera.. On the one hand, with sample representation relying on statistical features such as TF–IDF that are used in this study and by [21] or on the crafted ones by [30], such features show superiority over the embedding techniques used by [15,31,32], Goldani, Momtazi [23], and Samadi, Mousavian [22]. On the other hand, the designed deep learning-based classifiers based on deep and dense sequential networks outperform other classifiers in terms of extracting the hidden representative features as compared to CNN used by Wang [31], Goldani, Safabakhsh [32], and Samadi, Mousavian [22] and as compared to LSTM used by Long [15].

## 6. Conclusions

Automatic detection of fake news is an ongoing challenging problem in the real world. Existing solutions focus on using embedding techniques to extract the salient features. We hypothesize that the use of feature embedding techniques may not be the best approach for fake news detection as it does not necessarily contain semantic or sentiment patterns that make it significantly different from the real news. This is due to the fact that false news and legitimate news both have comparable language traits. In this study, a deep and dense ensemble model has been designed and developed for fake news detection to improve the detection accuracy of fake news. The study shows that, with proper representation and proper model design, the conventional features based on news content outperform the existing text embedding techniques. The proposed model was constructed in three phases. In the first phase, features were extracted from the news content, enriched using n-gram techniques, and then represented using the TF–IDF statistical technique. Multiple binary classifiers were designed based on the correctness of the news to extract the hidden features that represent the news well. The final classification was constructed using the multilayer perceptron by learning from the constructed deep ensemble model parameters. A multi-classifier was trained based on the outputs of the ensemble set of classifiers for decision-making to improve detection. The LIAR and ISOT datasets, which are commonly used in related work, were used in this study to validate the proposed model. The proposed model has been evaluated by comparing its performance with the related work. Results based on LIAR datasets, which are most challenging due to short news sentences and their multi-class nature, show the superiority of the proposed model compared to current state-of-the-art ones. The proposed model achieved 100% detection accuracy on the ISOT dataset, implying that it works more effectively and efficiently compared with existing solutions in short and long news type content, depending solely on content-based features.

This study is limited to content-based features, which may not be enough to improve the performance in short news, such as in social media, as shown by the LIAR dataset. A deep analysis of news origins and context should also be investigated for future work. As long as the news content is not wholly wrong, the conveyed message should be extracted first. We are working on integrating contextual features and extracting the features from other related sources, and the results will be in our future publications. Moreover, the proposed ensemble model in this study can be further improved by augmenting the existing predictive learners with learners trained based on features extracted using embedding techniques.

## Figures and Tables

**Table 1:** Number of Samples in the LIAR Dataset.

Group	Labels						Total
	Barely-True	Mostly-True	True	Half-True	Pants-Fire	False	
Training	1654	1962	1676	2114	839	1994	10,239
Validation	236	251	169	248	116	263	1283
Testing	212	241	207	265	92	249	1266

**Table 2:** Quantity of Samples in the ISOT Dataset.

	Labels	
	Fake	True
Total	23,481	21,417

**Table 3:** Classification Performance for LIAR Dataset.

Classifier	1st Stage Multiple Binary Classification				2nd Stage Multi-Class Classification			
	Accuracy	Precision	Recall	F1 Score	Accuracy Precision		Recall	F1 Score
Pants-fire	0.88	0.94	0.93	0.94	0.71	0.92	0.74	0.82
False	0.60	0.81	0.67	0.73	0.52	0.83	0.50	0.62
Barely-true	0.77	0.84	0.90	0.87	0.39	0.86	0.33	0.48
True	0.77	0.84	0.90	0.87	0.56	0.90	0.38	0.67
Half-true	0.73	0.80	0.88	0.84	0.44	0.78	0.40	0.53
Mostly-true	0.76	0.81	0.91	0.86	0.44	0.87	0.38	0.53
Average	0.75	0.84	0.87	0.85	**0.51**	**0.86**	**0.45**	**0.61**

**Table 4:** Classification Accuracy of the Models using LIAR dataset.

Author and Year	Features	Representation	Classifier	F1 Score
Wang [31], 2017	Text	Word2Vec	Hybrid CNN	27.01%
Long [15], 2017	Speaker profile	Weighted vectors	LSTM attention	41.5%
Goldani, Safabakhsh [32], 2021	Text	Glove.6B.300d	CNN with margin loss	40.58%
Goldani, Momtazi [23], 2021	Text	Word2Vec	CNN	39.50%
Hakak, Alazab [30], 2021	derived (Text + NER)	Statistical	RF	44.15%
Samadi, Mousavian [22], 2021	2021	Funnel	CNN	48.64%
The proposed model	Text	TF–IDF-IG	SDL–MLP	51.05%

**Table 5:** Classification Accuracy of the Proposed Model using ISOT Dataset.

Classifier	1st Stage Multiple Binary Classification				2nd Stage Multi-Class Classification			
	Accuracy	Precision	Recall	F1 Score	Accuracy	Precision	Recall	F1 Score
Real	99.94	100	99.89	99.94	100	100	100	100
Fake	99.94	99.89	100	99.95	100	100	100	100
Average	99.94	99.94	99.94	99.94	100	100	100	100

**Table 6:** Classification Accuracy of the Models using ISOT dataset.

Author and Year	Features	Representation	Classifier	F1 Score
Goldani, Safabakhsh [32], 2021	Text	Glove.6B.300d	CNN with margin loss	99.90%
Ahmed, Traore [24], 2017	Text	GloVe	LSVM	92.00%
Goldani, Momtazi [23], 2021	Text	Word2Vec	CNN	99.8%
Hakak, Alazab [30], 2021	derived (Text + NER)	Statistical	RF	100%
Samadi, Mousavian [22], 2021	Text	Funnel	CNN	99.96%
The proposed model	Text	TF–IDF-IG	SDL–MLP	100%

## Data Availability

The datasets that were used in this study are available online on the following links. 1. LIAR Dataset: https://www.cs.ucsb.edu/~william/data/liar_dataset.zip, accessed on 20 January 2022. 2. ISOT Dataset: https://paperswithcode.com/dataset/isot-fake-news-dataset, accessed on 5 April 2022.
